# A77 IMPLEMENTATION OF GUT LINK-IBS; RESULTS OF A SEMI-STRUCTURED INTERVIEW OF PRIMARY CARE PROVIDERS

**DOI:** 10.1093/jcag/gwab049.076

**Published:** 2022-02-21

**Authors:** S Sharma, C Heisler, J Jones, M J Stewart

**Affiliations:** 1 Gastroenterology, Dalhousie University, Halifax, NS, Canada; 2 Medicine, Dalhousie University, Halifax, NS, Canada; 4 Gastroenterology, Research Services, QEII Health Sciences Centre, Halifax, NS, Canada

## Abstract

**Background:**

The Division of Digestive Care and Endoscopy in Halifax, Nova Scotia has had longstanding challenges with GI referral volume outstripping divisional capacity resulting in limited access to specialist care. Many referrals for functional bowel disorders (FBD) are returned to referring providers. Although this helps rationalize limited system resources, it often impairs access to appropriate and necessary GI care. The co-development and implementation of clinical care pathways across gastroenterology and primary care may help to improve access to high-quality GI care.

**Aims:**

The project aimed to engage primary healthcare providers (PHCPs) to identify environmental and behavioral barriers and facilitators for managing undifferentiated lower GI disorders in primary care. Data generated from stakeholder engagement will be used to develop, implement, and evaluate strategies for referral and management of FBD. A real-world, functional clinical care pathway that supports the implementation of evidence-based practices in the diagnosis and management of functional GI conditions within primary care will enhance care and timely access to specialist.

**Methods:**

This is a qualitative study using semi-structured interviews of PHCPs working in Nova Scotia. Interview questions were developed and guided by the evidence-based implementation science frameworks. Physicians were recruited through existing primary care networks. Participants were offered a Zoom™virtual semi-structured interview. A brief intake questionnaire was administered to collect baseline demographics. Interviews were recorded and transcribed for data analysis. Data were categorized into coding schemes and themes were created using an inductive coding approach.

**Results:**

As of October 2021, 9 interviews have been conducted. Average participant age was 44 years, with the majority identifying practice in a group or collaborative care setting (n=7, 78%). Five worked in urban practice settings and the remainder in rural areas. Preliminary major themes included:

1. A lack of satisfaction with access to gastrointestinal care, with most physicians noting it to be worse than access to other specialist services.

2. Management of FBDs were felt to be within the scope of primary care practice

3. Access to diagnostic tests like fecal calprotectin with appropriate education on its use as a diagnostic tool would be useful.

4. PHCP’s suggested care pathways be easy to use, require minimal time, and ideally be implemented within their pre-existing EMR or in paper form.

**Conclusions:**

PHCPs acknowledge a significant burden of undifferentiated lower GI complaints in their practice and poor access to gastroenterology services. All participants were open to helping develop and use a clinical care pathway for the investigation and management of undifferentiated lower GI symptoms. Data collection and analysis are ongoing.

**Funding Agencies:**

None

